# Non-invasive opportunistic screening for diabetes mellitus: an interpretable stacking ensemble framework

**DOI:** 10.1038/s41598-026-57865-9

**Published:** 2026-06-16

**Authors:** Lin Zhang, Junming Xu, Weigang Wang, Rui Chang, Linghua Wang

**Affiliations:** 1 Zhejiang Hospital of Integrated Traditional Chinese and Western Medicine, Hangzhou, 310003 China; 2 Department of Statistics and Data Science, Zhejiang Gongshang University, Hangzhou, 310018 China; 3 Jining No.1 People’s Hospital, Jining, 272100 China

**Keywords:** Diabetes mellitus, Opportunistic screening, Interpretable stacking, In Silico simulation, SMOTENC, Health care, Mathematics and computing

## Abstract

Opportunistic screening for type 2 diabetes offers a potentially accessible approach to preliminary case detection without relying on invasive testing. In this study, we developed a heterogeneous Stacking ensemble model (Task C) using exclusively non-invasive demographic, lifestyle, medical-history, and symptom-based features. The model prioritized sensitivity, achieving a Recall of 0.9267, while showing modest discriminative performance (AUC = 0.5515), low specificity (0.1106), and moderate probability calibration (Brier Score = 0.2482). Targeted simulation analyses revealed that adjusting the top three modifiable behavioral factors captured approximately 85.5% of the reduction in model-estimated screening probability observed under the all-six-factor adjustment. Individual-level case simulation illustrated a stepwise reduction in model-estimated screening probability under increasingly comprehensive hypothetical adjustments. Decision curve analysis suggested potential screening utility mainly within the lower-threshold range. These findings suggest that the proposed ensemble may serve as a technically feasible and interpretable tool for preliminary non-invasive diabetes case-finding, while providing hypothesis-generating insights into modifiable factors for future validation.

## Introduction

Diabetes Mellitus (DM) represents a critical global public health challenge, with its prevalence rising significantly, particularly among younger demographics^1^. Because pharmacological interventions alone are insufficient to reverse this trend, clinical focus has increasingly shifted from reactive treatment to proactive prevention. For Type 2 Diabetes, which is closely associated with modifiable lifestyle factors, large-scale opportunistic screening coupled with lifestyle management forms the cornerstone of the “Three-Level Prevention” strategy.

Despite the potential of AI in medical diagnostics^2,3^, three major translational barriers hinder its real-world screening deployment: (1) Class Imbalance: Diabetic patients are typically the minority in screening cohorts, causing standard models to favor global accuracy over sensitivity, thereby increasing false-negative rates^4^; (2) Data Heterogeneity: Models trained on single-center data often lack generalizability across diverse demographic populations^5^; (3) Algorithmic Opacity: The “black box” nature of complex models hinders clinical trust and limits actionable preventive guidance^6^.

To overcome these barriers, recent research has shifted toward knowledge-augmented modeling strategies, which combine data-driven algorithms with clinical expertise to improve dimensionality control and robustness. As demonstrated by recent studies^7,8^, integrating domain expertise with explainable AI can effectively mitigate high-dimensional overfitting and enhance generalizability across varied clinical settings. However, a gap remains for practical frameworks capable of autonomously filtering and prioritizing modifiable lifestyle factors for high-sensitivity screening within an integrated decision system.

Addressing this gap, the present study constructs a framework bridging data mining with knowledge-guided recommendations. We explicitly confine our scope to cross-sectional, non-invasive opportunistic screening—distinct from clinical diagnosis (which relies on invasive biochemical markers like HbA1c) and true longitudinal early-risk prediction. The principal contributions are summarized as follows:

- *A Heterogeneous Stacking Framework with Feature Augmentation:* We propose a hybrid modeling strategy combining unsupervised mining and supervised ensembles. By explicitly embedding latent patient subgroup structures as “distance meta-features,” this data-driven approach enhances the model’s capability to capture pathological heterogeneity and helps reduce the risk of high-dimensional overfitting.
- *A Hybrid Sampling Strategy for Mixed-Type Data:* We introduce the SMOTENC algorithm to resolve interpolation artifacts in medical datasets containing both nominal and continuous variables^9^. This strategy mitigates class imbalance while strictly preserving the authentic distribution of cross-sectional features.
- *From Screening to Knowledge-Validated Recommendation:* Moving beyond simple SHAP attribution^10^, we establish a practical decision chain. By constraining algorithmic rankings with medical domain knowledge—specifically filtering out immutable factors (e.g., age, genetics)—we propose a “Top-3 Screening-Oriented Simulation” strategy. Its utility is evaluated via *in silico* counterfactual simulations and Decision Curve Analysis (DCA), demonstrating a pragmatic paradigm that translates algorithmic screening into screening-oriented behavioral insights without asserting direct clinical causality.

## Related work

### Machine learning in opportunistic screening and risk stratification

The increasing availability of healthcare data has made machine learning pivotal in chronic disease prevention and opportunistic screening. Early research primarily relied on traditional statistical models, such as Logistic Regression. While offering inherent interpretability, these models often struggle to capture complex, non-linear pathological interactions^11^. In contrast, non-linear models like Support Vector Machines (SVM) and Deep Neural Networks (DNN) improve fitting accuracy but inevitably introduce the “bias-variance tradeoff”^12^. Furthermore, a persistent challenge in this domain is “class imbalance,” where single models frequently fail to appropriately balance specificity and sensitivity^13^. This underscores the critical need for robust algorithmic methods tailored to real-world disease prevalence.

### Ensemble learning and stacking algorithms

To mitigate the limitations of single models, ensemble learning constructs robust classifiers. Homogeneous ensemble strategies, such as Random Forest and XGBoost, have demonstrated strong predictive performance^14,15^. However, these approaches are often constrained by the inherent inductive bias of a single algorithm family. Conversely, Stacking integrates diverse algorithms to overcome this limitation. As Kumari et al.^16^ confirmed, Stacking effectively combines linear and non-linear models. Particularly for imbalanced data, Hsieh et al.^17^ demonstrated that a hierarchical Stacking architecture with weighted base learners can significantly improve the recall rate for minority class patients.

### Explainable artificial intelligence (XAI) and knowledge-augmented modeling

The “black box” nature of complex models often hinders clinical trust. The SHAP (SHapley Additive exPlanations) framework provides a robust mechanism to address this, utilizing game theory for feature importance ranking^10^. Research by El-Sappagh et al.^18^ indicates that SHAP can effectively quantify marginal contributions.

However, purely data-driven XAI often fails to differentiate between predictive correlations and clinically actionable targets. To bridge this gap, recent literature emphasizes medical-knowledge-assisted hybrid modeling. Samadi et al.^7^ demonstrated that training strategies incorporating clinical expertise can mitigate high-dimensional overfitting. Furthermore, integrating domain knowledge with SHAP-based analysis improves cross-population generalizability^8^, and recent frameworks leverage large language models to augment interpretability in critical care^19^. These advancements underscore the necessity of constraining algorithmic outputs with clinical realities.

Integrating XAI with domain knowledge bridges the gap between screening and practical decision support. As Li et al.^20^ noted, utilizing high-weight features identified by SHAP can inform personalized preventive strategies, aligning with the lifestyle management philosophy emphasized in the Daqing Diabetes Prevention Study^21,22^.

### Summary and research gap

A review of the literature reveals several persistent methodological gaps: (1) Homogeneity Limitation: Current models often struggle to balance linear and non-linear feature representations. (2) Imbalance Handling and Performance Trade-off: There is a lack of targeted strategies that maximize screening recall while explicitly managing the clinical burden of false positives. (3) Disconnect between Screening and Knowledge-Guided Recommendations: Existing models frequently fail to translate model outputs into screening-oriented, clinically interpretable information, lacking mechanisms to filter correlational features through a medical-knowledge lens.

This study aims to bridge these gaps using an interpretable Stacking framework, providing screening-oriented simulation support constrained by clinical plausibility.

## Data preprocessing and feature engineering

### Data sources and feature description

#### Dataset overview

This study used a publicly available cross-sectional cohort dataset comprising 1,879 individuals, including 752 individuals labeled as diabetic (Label = 1) and 1,127 non-diabetic individuals (Label = 0). The dataset contains 43 candidate variables spanning demographic characteristics, lifestyle factors, vital signs, and biochemical indicators, with a positive-to-negative ratio of approximately 1:1.5, reflecting a mild class imbalance relevant to opportunistic screening applications.

As a cross-sectional dataset, it is appropriate for evaluating opportunistic diabetes screening and case-finding rather than longitudinal early-risk prediction. While the original class distribution was preserved to support realistic model evaluation, the cohort is single-center, which limits immediate generalizability. Future studies should validate these findings across independent, multi-center populations to assess cross-population robustness.

#### Hierarchical feature stratification and task definition

To evaluate the applicability of the proposed framework across different healthcare settings and the practical value of low-cost screening, we constructed three progressively constrained task scenarios: Task A, Task B, and Task C. These tasks were stratified by invasiveness, cost, and accessibility of feature acquisition, forming a continuum from hospital-based diagnostic confirmation to community- or home-based self-assessment (Table 1).

*Task A: Diagnostic Classification Benchmark (Biochemical Markers). *Hospital-based diagnostic confirmation using all 43 variables, including biochemical markers such as FPG and HbA1c. Task A serves as a benchmark for maximum discriminative performance and is not intended for early-risk prediction.

*Task B: Primary Care Screening Scenario (Physical Examination). *Non-invasive primary-care screening using demographics, lifestyle questionnaires, and basic physical measurements (e.g., BP, BMI). Designed to evaluate whether simple objective measures improve risk stratification over questionnaire-only assessment.

*Task C: Non-invasive Opportunistic Screening Scenario (Self-reported Indicators). *Fully non-invasive, cross-sectional screening using 21 self-reported variables, including demographics, family history, mild concurrent symptoms, previous pre-diabetes, and lifestyle indices. Variables were selected based on their relevance to cross-sectional opportunistic screening; removed features included late-stage or complication-related symptoms, environmental exposure variables, and indirect health-management behaviors. Task C is intended for case-finding; some features are proximal to current disease status, so it should not be interpreted as a longitudinal early-risk prediction model or a stand-alone diagnostic instrument. The primary goal is to identify individuals who may benefit from confirmatory biochemical testing.

**Table 1:** Hierarchical feature stratification and representative variables for each task.

Category	Representative Variables	Task A	Task B	Task C
		(Diagnosis)	(Clinical)	(Self-reported)
Basic Info.	Age, Gender, Education, Family History, Previous Pre-Diabetes	*\checkmark*	*\checkmark*	*\checkmark*
Lifestyle	Smoking, Alcohol, Activity, DietQuality, SleepQuality	*\checkmark*	*\checkmark*	*\checkmark*
Symptoms	Polyuria, Polydipsia, Fatigue	*\checkmark*	*\checkmark*	*\checkmark*
Physical Signs	Systolic BP, Diastolic BP	*\checkmark*	*\checkmark*	–
Biochemical	HbA1c, FPG, LDL/HDL Cholesterol	*\checkmark*	–	–
*Feature Count/Cost Level*		43/High	35/Low	21/None

BP Blood Pressure; FPG Fasting Plasma Glucose. Representative variables are examples rather than an exhaustive feature list.

#### Rationale for feature stratification

The three task scenarios were designed to evaluate model performance under progressively constrained feature accessibility, ranging from fully diagnostic (Task A) to non-invasive opportunistic screening (Task C). This design allows assessment of whether increasingly accessible feature sets can still support high-sensitivity identification of individuals who may require confirmatory biochemical testing.

### Data preprocessing

#### Data cleaning and train–test partitioning

Non-predictive identifiers, including *PatientID* and *DoctorInCharge*, were removed before model development, and the target variable was defined as *Diagnosis*. The dataset was divided into a training set and an independent held-out test set at an 8:2 ratio using stratified sampling, thereby preserving the original class distribution in both subsets.

To prevent information leakage, all data-dependent preprocessing parameters were estimated exclusively from the training data. Missing values were imputed using medians calculated from the training set, and the same imputation parameters were then applied to the held-out test set. The held-out test set was reserved strictly for final model evaluation and was not used for model tuning, resampling, feature augmentation, threshold selection, or hyperparameter optimization.

#### Correlation analysis

**Fig. 1:**
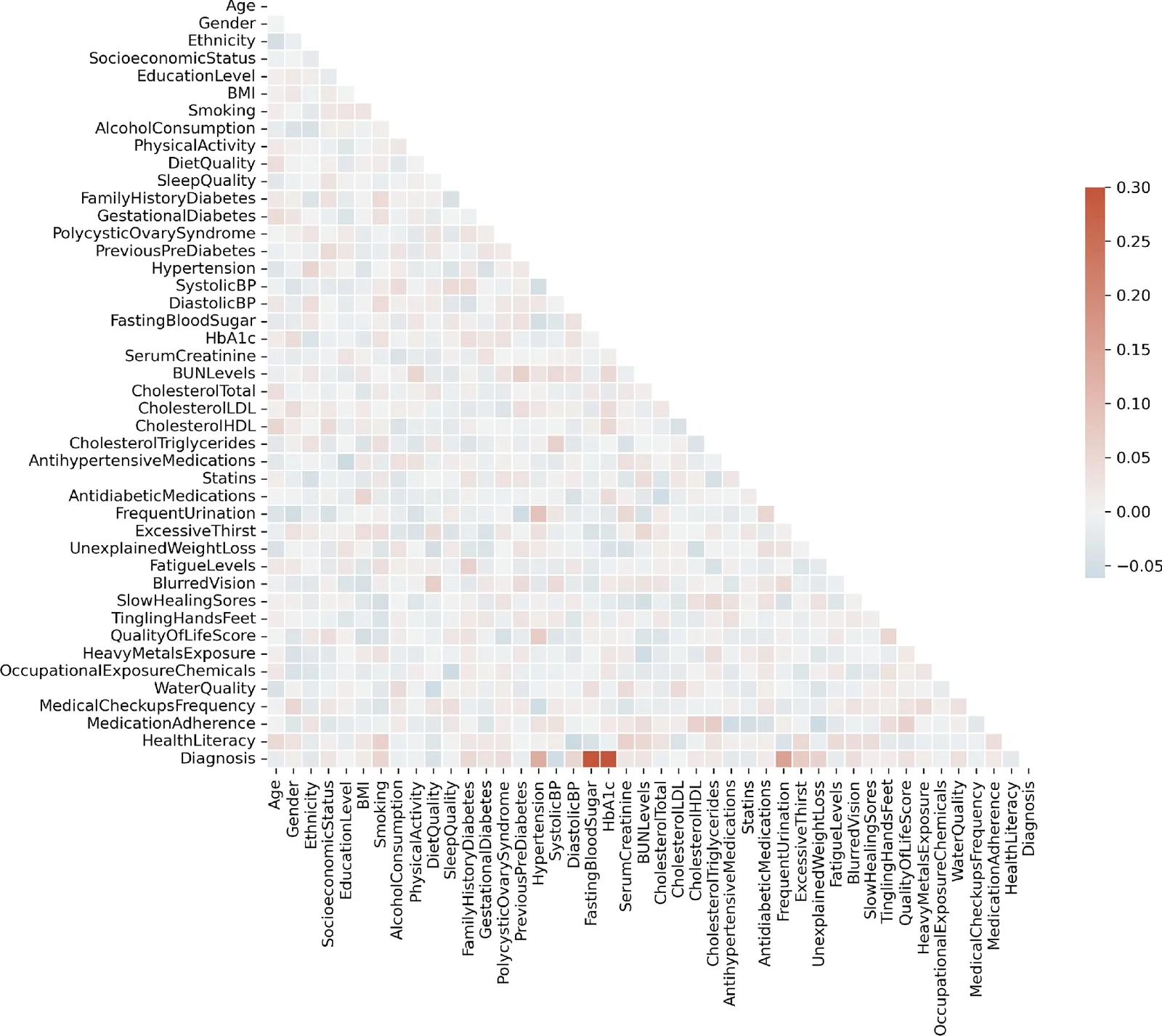
Spearman rank correlation heatmap.

Before model training, Spearman’s rank correlation analysis was conducted to examine pairwise associations among candidate predictors and the outcome variable (Fig. 1). Although several non-invasive variables showed weak marginal correlations with *Diagnosis*, they were retained to preserve clinically relevant information and enable the Stacking model to capture potential high-order non-linear interactions among demographic, lifestyle, medical-history, and symptom-related factors.

#### Standardization of continuous variables

Given the mixed-type structure of the dataset, continuous and categorical variables were processed separately. Continuous variables were standardized using Z-score normalization, whereas categorical variables were kept in their original encoded form. During cross-validation, the scaler was fitted within each training fold and then applied to the corresponding validation fold. For the final held-out evaluation, the scaler was fitted on the full training set and applied to the test set.

#### Handling class imbalance with SMOTENC

Class imbalance in the Task C dataset was addressed using the Synthetic Minority Over-sampling Technique for Nominal and Continuous features (SMOTENC). SMOTENC was selected because the dataset contains both continuous variables and nominal or binary variables, and the algorithm can generate synthetic minority-class samples while preserving the discrete structure of categorical predictors.

Before applying SMOTENC, variables were explicitly classified according to their measurement type. In each task-specific feature matrix, nominal or binary variables, including demographic variables (e.g., *Gender* and *Ethnicity*) and binary medical-history or symptom indicators, were treated as categorical predictors. Continuous variables, including physiological and lifestyle-related measures such as *Age*, *BMI*, *AlcoholConsumption*, *PhysicalActivity*, *DietQuality*, and *SleepQuality*, were treated as continuous predictors. Continuous variables were standardized before resampling, whereas categorical variables were retained in their original encoded form.

To prevent information leakage, SMOTENC was applied only to the training data. During cross-validation, resampling was performed separately within each training fold after fold-specific preprocessing. For final model training, SMOTENC was applied only to the full training set. The validation folds and the independent held-out test set were never resampled, thereby preserving their original class distributions for unbiased performance evaluation.

For comparison, ADASYN was also evaluated as a conventional oversampling alternative. Because ADASYN treats all predictors as continuous during interpolation, it generated 97 invalid values for the binary *Gender* feature, indicating that naive oversampling may produce clinically meaningless intermediate states. This empirical finding further supported the use of SMOTENC for mixed-type medical predictors.

#### Cluster-distance-based feature augmentation and sensitivity analysis

To capture latent non-linear structure in the feature space, K-Means-based distance features were incorporated into the optimized Stacking model. After fold-specific preprocessing and SMOTENC resampling, K-Means was fitted on the resampled training data. The Euclidean distances from each sample to the learned centroids were then appended to the processed feature matrix as continuous meta-features. For validation and test samples, the centroids learned from the corresponding training data were used to compute distance features. This design provided non-linear structural information without assigning samples to hard clusters.

**Fig. 2:**
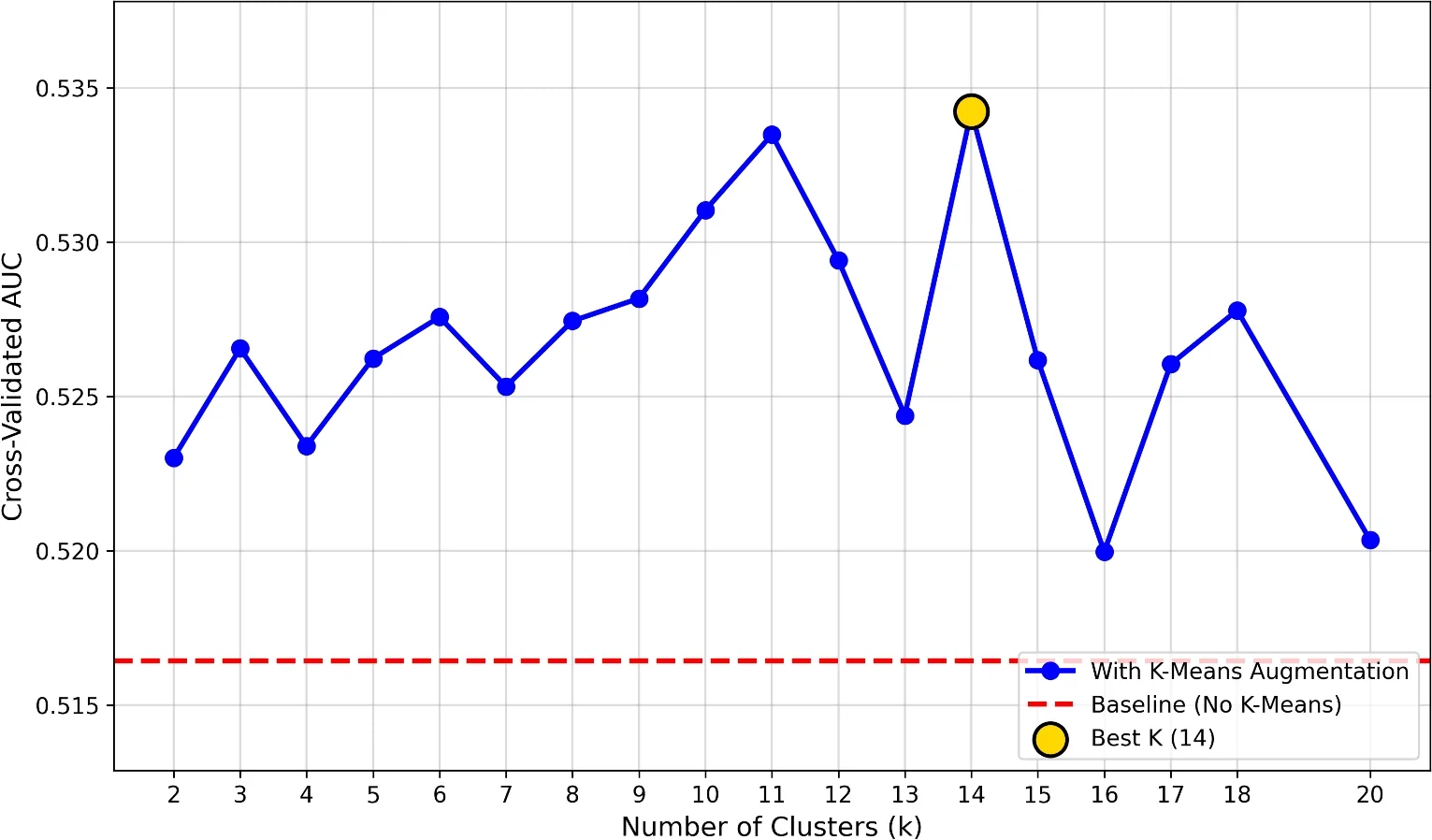
Sensitivity Analysis of K-Means Feature Augmentation. The cross-validated AUC demonstrates that introducing distance meta-features consistently outperforms the unaugmented baseline (red dashed line). The optimal cluster number (*k=14*) was selected to maximize predictive performance while maintaining feature parsimony.

The optimal number of clusters (*k*) was determined using training-set out-of-fold predictions. Candidate values of *k* from 2 to 20 were evaluated, with K-Means fitted within the training folds after preprocessing and SMOTENC resampling. Based on the sensitivity analysis, *k=14* was selected and fixed for the final Task C Stacking model before hyperparameter optimization and held-out test-set evaluation (Fig. 2).

## Screening model construction based on heterogeneous stacking

### Stacking architecture design

**Fig. 3:**
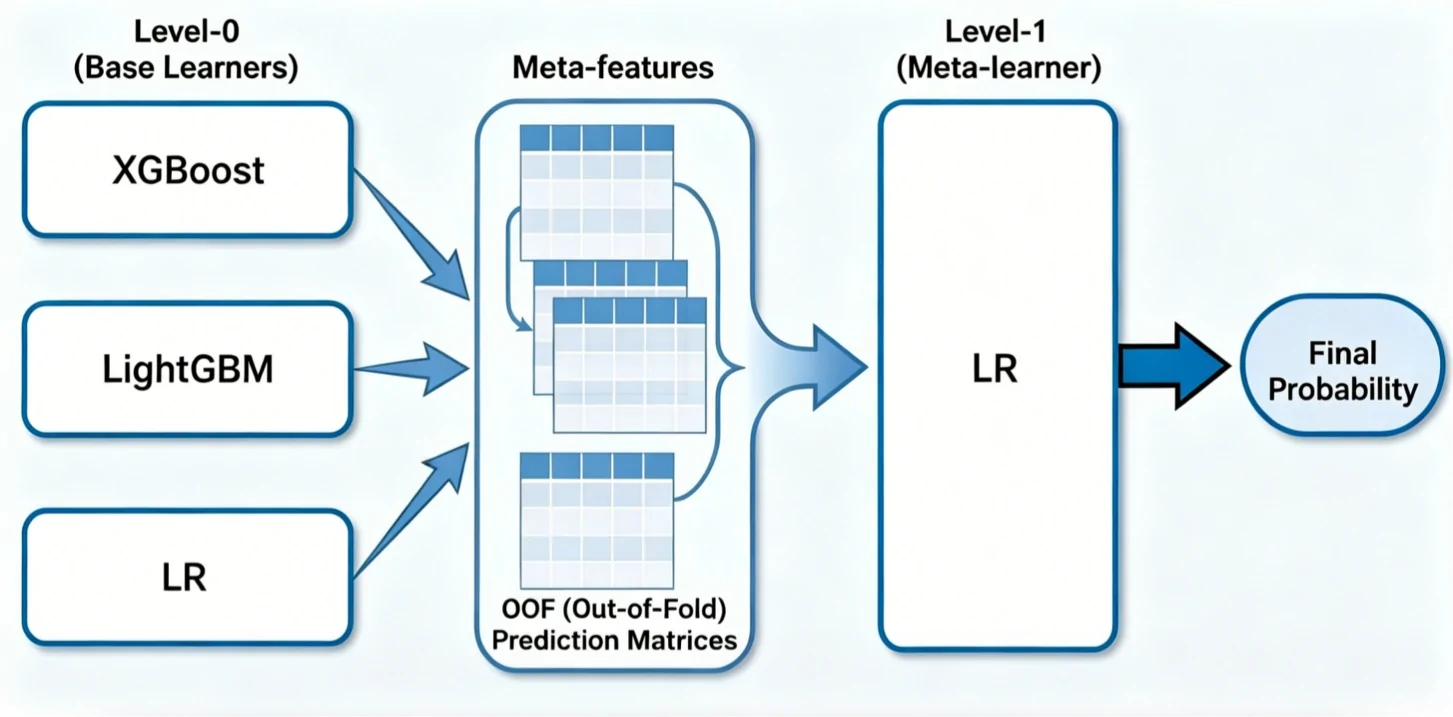
Topolog of the optimized Stacking ensemble model. The Level-0 layer integrates two gradient-boosting models, XGBoost and LightGBM, with Logistic Regression to capture complementary non-linear and linear patterns. The Level-1 layer uses Logistic Regression as the meta-learner to aggregate out-of-fold predictive probabilities and generate the final screening probability.

Stacking is a hierarchical ensemble learning framework in which a meta-learner integrates the predictive outputs of multiple base learners. In this study, we developed a two-layer heterogeneous Stacking architecture (Fig. 3) to combine models with different inductive biases. The input to the Stacking framework consisted of the preprocessed feature matrix augmented with K-Means distance-based meta-features, allowing the model to use both original clinical information and latent structural representations.

The Level-0 layer was designed to capture complementary predictive patterns from the data, whereas the Level-1 layer was kept deliberately simple to reduce the risk of secondary overfitting. This design is particularly suitable for opportunistic diabetes screening, where both non-linear risk interactions and stable probability aggregation are required.

#### Level-0: heterogeneous base learners

The Level-0 layer consisted of XGBoost, LightGBM, and Logistic Regression. XGBoost and LightGBM were selected because gradient-boosting models are well suited for tabular clinical data and can capture complex non-linear feature interactions. Logistic Regression was included as a linear baseline to provide a stable and interpretable probability component. By combining tree-based boosting models with a linear model, the Level-0 layer generated diverse predictive representations that could be further integrated by the meta-learner.

#### Level-1: logistic regression meta-learner

Logistic Regression was used as the Level-1 meta-learner. Because the Level-0 learners already included strong non-linear models, using a simple linear meta-learner helped reduce the risk of overfitting at the second stage. The meta-learner received the out-of-fold predicted probabilities from the Level-0 models and learned their optimal linear combination. This strategy enabled robust aggregation of complementary model outputs while maintaining a transparent final decision layer for screening-oriented risk estimation.

#### Training, validation, and reproducibility

The Task C Stacking model was trained using 5-fold cross-validation to generate out-of-fold predicted probabilities from the Level-0 learners, which were then used as input to the Level-1 Logistic Regression meta-learner. All data-dependent preprocessing steps, including standardization, SMOTENC resampling, and K-Means distance augmentation, were performed strictly within the training folds. The held-out test set remained completely independent and was used only for final performance evaluation.

Data partitioning was performed with a fixed random seed (42). The dataset was divided into 80% training and 20% held-out test samples using stratified sampling to preserve the original class distribution. Threshold selection, feature augmentation, and hyperparameter tuning were conducted exclusively on the training data or training folds. Key model hyperparameters were fixed before held-out test-set evaluation and are summarized in Table 2.

**Table 2:** Key Hyperparameters of the Task C Stacking Model.

Layer	Model	Key Hyperparameters
Level-0	XGBoost	n_estimators=100, max_depth=3, learning_rate=0.03; subsample=0.8, colsample_bytree=0.8; min_child_weight=5, reg_alpha=0.1, reg_lambda=1.0
Level-0	LightGBM	n_estimators=100, learning_rate=0.04, max_depth=2; num_leaves=4, min_child_samples=30; reg_alpha=0.1, reg_lambda=1.0
Level-0	LR	max_iter=1000, C=0.2, penalty=L2, solver=lbfgs
Level-1	LR	max_iter=2000, C=0.1, penalty=L2, solver=lbfgs

### Evaluation metrics

To evaluate model performance across diverse clinical dimensions, we utilized a combination of discriminative, calibration, and clinical utility metrics.

#### Classification metrics: sensitivity and specificity trade-offs

Given the asymmetry of misclassification costs in public health screening—where a false negative (missed diagnosis) risks severe complications, while a false positive generates unnecessary healthcare burdens via re-examination—we adopted a sensitivity-driven evaluation approach. Consequently, Recall (Sensitivity) was prioritized to maximize the capture of potential patients. However, to explicitly address the practical clinical implications of over-referrals, Specificity was formally included to quantify the model’s false-positive burden. Complementarily, Precision and F1-score were employed to assess the reliability of warning signals and the overall balance between positive and negative classes.

To ensure the statistical robustness of our findings, 95% Confidence Intervals (CIs) were calculated for all primary metrics, including AUC, Recall, Precision, and F1-score. The operating threshold was selected from training-set out-of-fold predictions under a sensitivity-prioritized criterion, with the target Recall set to at least 0.90. The selected threshold was then fixed before evaluation on the independent held-out test set. Furthermore, calibration analyses, including calibration curves and the Hosmer-Lemeshow test, were incorporated to assess the agreement between the predicted screening probabilities and the observed empirical frequencies.

#### Generalization metric: AUC

To assess overall discriminative performance independent of specific classification thresholds, we utilized the Area Under the Receiver Operating Characteristic Curve (AUC) as the primary metric. Unlike single-cutoff metrics, AUC provides a threshold-invariant evaluation. Particularly in the class-imbalanced scenarios of this study, AUC mitigates the evaluation bias typically dominated by the majority class (healthy individuals), thereby offering an objective measure of the model’s discriminative power across a continuous range of clinical thresholds.

#### Clinical utility assessment: decision curve analysis

While traditional statistical metrics evaluate predictive correctness, they do not quantify the clinical consequences or the false-positive burden inherent in public health screening. To address this limitation, we applied Decision Curve Analysis (DCA). DCA transforms the cost of false positives into a deduction from the benefit of true positives by introducing a threshold probability ($p_t$). The Net Benefit (NB) is mathematically defined as:

1 $$\begin{aligned} \text {Net Benefit} = \frac{\text {True Positives}}{N} - \frac{\text {False Positives}}{N} \times \frac{p_t}{1 - p_t} \end{aligned}$$

where *N* is the total number of samples. The weighting term $\frac{p_t}{1 - p_t}$ represents the relative harm of a false positive versus a false negative. This analysis quantifies the net clinical benefit of the model compared to default strategies (e.g., treat-all or treat-none) across various risk thresholds, explicitly translating algorithmic probabilities into practical decision-making utility.

## Experimental results and analysis

### Comparative analysis of model performance

#### Performance comparison: stacking vs. single models

The core performance metrics across the three tiered tasks are detailed in Table 3.

- *Task A (Diagnostic Classification Benchmark):* In the feature-complete Task A, all models achieved substantially higher discriminative performance than in the non-invasive scenarios, reflecting the strong predictive contribution of biochemical diagnostic markers. The optimized Stacking framework achieved an AUC of 0.9500 (95% CI: 0.9225–0.9714) and a Recall of 0.9067. Although GBDT and LightGBM yielded slightly higher AUC values in this diagnostic benchmark, the Task A results mainly served as an upper-bound reference for evaluating the performance trade-offs introduced by progressively removing biochemical and physical examination features.
- *Task B and C (Non-invasive Opportunistic Screening): Performance Trade-offs.* In the non-invasive opportunistic screening scenario (Task C), overall discriminative performance decreased due to the absence of biochemical markers. The optimized Stacking model achieved an AUC of 0.5515 (95% CI: 0.4941–0.6079) and a high Recall of 0.9267, prioritizing the identification of potentially undiagnosed individuals. This trade-off came with low specificity (0.1106, 95% CI: 0.0703–0.1535) and moderate Precision (0.4088, 95% CI: 0.3588–0.4631), reflecting a substantial number of false positives. However, in opportunistic screening, false positives can be resolved by follow-up confirmatory biochemical testing, whereas false negatives carry a higher clinical risk. The Brier Score of the Stacking model was 0.2482, indicating moderate probability calibration under the non-invasive feature setting.

**Table 3:** Screening Performance of the Evaluated Models Across Three Tiered Scenarios. The proposed Stacking framework achieves a favorable balance, maintaining high sensitivity and moderate probability calibration (Brier Score) even as feature availability is progressively constrained in Task C.

Scenario	Model	AUC	Recall	Precision	Specificity	F1-Score	Brier Score
Task A *(Benchmark)*	Stacking	0.9500 (0.9225–0.9714)	0.9067 (0.8560–0.9496)	0.8553 (0.7962–0.9085)	0.8982 (0.8571–0.9343)	0.8803 (0.8391–0.9155)	0.0717 (0.0558–0.0916)
	SVM	0.9122 (0.8764–0.9400)	0.9333 (0.8889–0.9708)	0.6393 (0.5758–0.7020)	0.6504 (0.5864–0.7124)	0.7588 (0.7093–0.8060)	0.1114 (0.0931–0.1335)
	GBDT	**0.9620** (0.9392–0.9802)	0.9067 (0.8561–0.9504)	0.9252 (0.8815–0.9662)	0.9513 (0.9209–0.9774)	0.9158 (0.8803–0.9450)	0.0636 (0.0480–0.0838)
	LightGBM	0.9615 (0.9386–0.9795)	**0.9467** (0.9071–0.9790)	**0.9342** (0.8933–0.9699)	**0.9558** (0.9273–0.9815)	**0.9404** (0.9117–0.9639)	**0.0587** (0.0437–0.0774)
	XGBoost	0.9576 (0.9326–0.9780)	0.9133 (0.8636–0.9554)	0.9257 (0.8794–0.9655)	0.9513 (0.9196–0.9774)	0.9195 (0.8859–0.9484)	0.0646 (0.0493–0.0840)
	AdaBoost	0.9466 (0.9188–0.9667)	0.9267 (0.8823–0.9630)	0.8176 (0.7558–0.8757)	0.8628 (0.8189–0.9052)	0.8688 (0.8259–0.9044)	0.2346 (0.2334–0.2359)
	LR	0.9121 (0.8779–0.9386)	0.9133 (0.8633–0.9562)	0.6432 (0.5772–0.7062)	0.6637 (0.5982–0.7218)	0.7548 (0.7021–0.8000)	0.1159 (0.0985–0.1376)
Task B *(Primary Care)*	Stacking	0.5434 (0.4826–0.6054)	0.8400 (0.7793–0.8993)	0.4038 (0.3522–0.4626)	**0.1770** (0.1266–0.2279)	0.5455 (0.4913–0.6013)	**0.2484** (0.2431–0.2539)
	SVM	0.5382 (0.4786–0.5986)	0.9467 (0.9086–0.9810)	**0.4226** (0.3742–0.4792)	0.1416 (0.0965–0.1880)	**0.5844** (0.5348–0.6364)	0.2512 (0.2385–0.2651)
	GBDT	0.5446 (0.4894–0.6057)	**0.9533** (0.9150–0.9865)	0.3939 (0.3444–0.4463)	0.0265 (0.0087–0.0495)	0.5575 (0.5060–0.6090)	0.2499 (0.2405–0.2590)
	LightGBM	0.5430 (0.4846–0.6035)	0.9400 (0.8985–0.9790)	0.3972 (0.3475–0.4482)	0.0531 (0.0261–0.0844)	0.5584 (0.5062–0.6110)	0.2513 (0.2413–0.2609)
	XGBoost	**0.5538** (0.4925–0.6149)	0.9333 (0.8896–0.9714)	0.4000 (0.3501–0.4551)	0.0708 (0.0400–0.1046)	0.5600 (0.5063–0.6122)	0.2488 (0.2384–0.2584)
	AdaBoost	0.5258 (0.4674–0.5858)	0.9400 (0.9000–0.9755)	0.4029 (0.3522–0.4546)	0.0752 (0.0431–0.1106)	0.5640 (0.5124–0.6139)	0.2493 (0.2484–0.2500)
	LR	0.5536 (0.4934–0.6140)	0.9200 (0.8734–0.9618)	0.4083 (0.3599–0.4613)	0.1150 (0.0739–0.1602)	0.5656 (0.5127–0.6175)	0.2537 (0.2409–0.2668)
Task C *(Opportunistic)*	Stacking	0.5515 (0.4941–0.6079)	0.9267 (0.8823–0.9662)	**0.4088** (0.3588–0.4631)	**0.1106** (0.0703–0.1535)	**0.5673** (0.5169–0.6193)	0.2482 (0.2454–0.2509)
	SVM	0.5435 (0.4836–0.5991)	**0.9400** (0.9006–0.9737)	0.4017 (0.3517–0.4554)	0.0708 (0.0385–0.1046)	0.5629 (0.5101–0.6166)	0.2518 (0.2413–0.2632)
	GBDT	0.5694 (0.5127–0.6303)	0.9200 (0.8766–0.9615)	0.4000 (0.3495–0.4529)	0.0841 (0.0493–0.1218)	0.5576 (0.5052–0.6094)	0.2450 (0.2364–0.2532)
	LightGBM	**0.5754** (0.5209–0.6355)	0.9200 (0.8749–0.9597)	0.4023 (0.3517–0.4564)	0.0929 (0.0566–0.1327)	0.5598 (0.5063–0.6124)	**0.2446** (0.2342–0.2542)
	XGBoost	0.5655 (0.5080–0.6222)	0.9200 (0.8750–0.9636)	0.4000 (0.3479–0.4519)	0.0841 (0.0502–0.1222)	0.5576 (0.5031–0.6077)	0.2455 (0.2358–0.2551)
	AdaBoost	0.5453 (0.4845–0.6070)	0.9133 (0.8645–0.9572)	0.4041 (0.3519–0.4575)	0.1062 (0.0694–0.1485)	0.5603 (0.5054–0.6124)	0.2497 (0.2476–0.2521)
	LR	0.5562 (0.4939–0.6151)	0.9333 (0.8936–0.9695)	0.4023 (0.3516–0.4553)	0.0796 (0.0456–0.1168)	0.5622 (0.5094–0.6139)	0.2487 (0.2396–0.2581)

#### Calibration assessment of the task C model

**Fig. 4:**
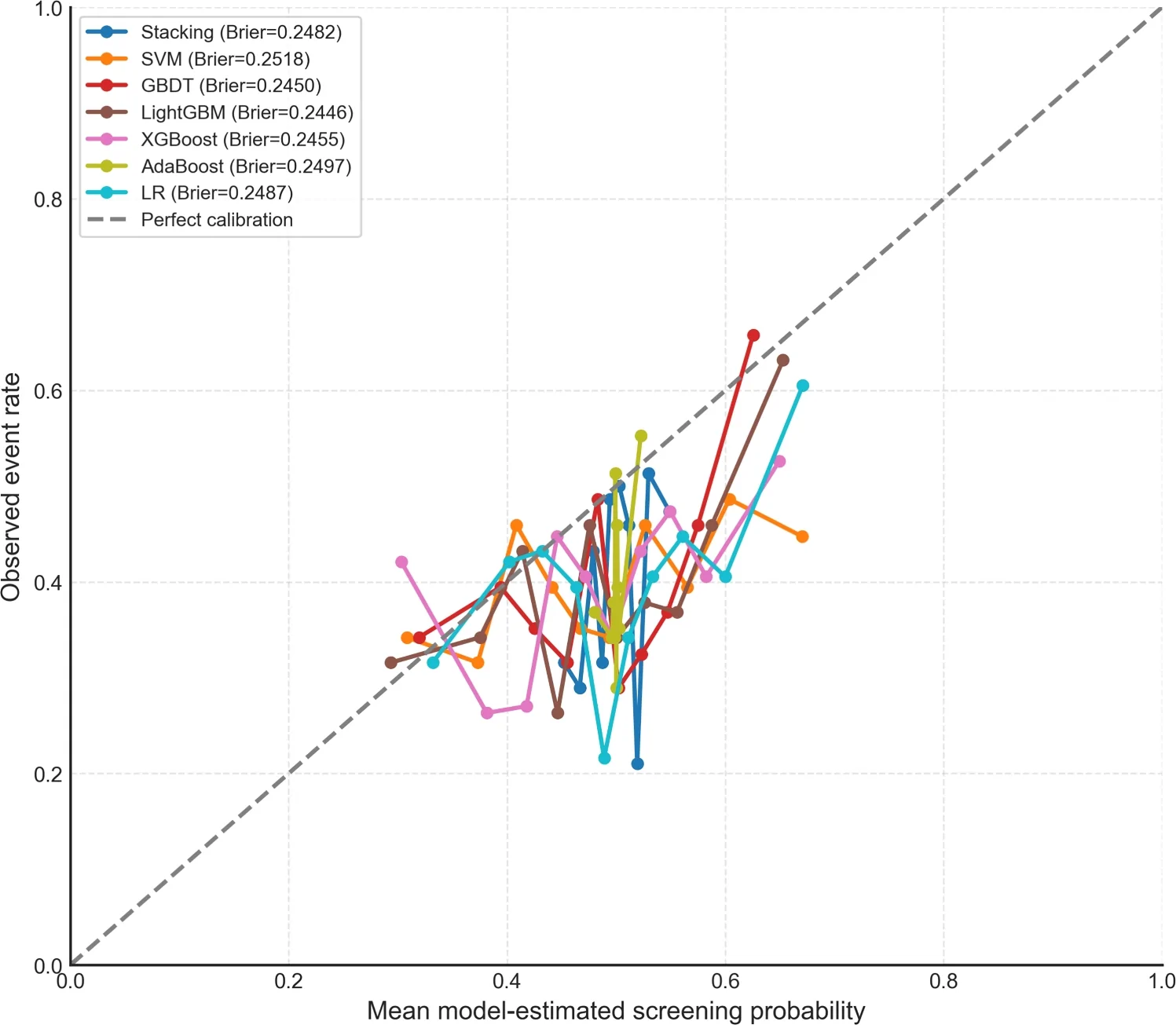
Calibration Curves of the Evaluated Models in Task C. The dashed line represents perfect calibration. The Brier Score and Hosmer–Lemeshow result of the main Stacking model are reported in the text and accompanying calibration table.

To evaluate the agreement between model-estimated screening probabilities and observed outcomes in the non-invasive opportunistic screening scenario (Task C), we generated a calibration curve for the Stacking model (Figure 4). The Brier Score was 0.2482, indicating moderate probability calibration. The Hosmer–Lemeshow goodness-of-fit test further quantified calibration across 10 risk groups. For the Stacking model, the test statistic was $\chi ^2 = 29.532$ with 8 degrees of freedom (*p = 0.0003*), demonstrating a statistically significant deviation from perfect calibration.

These results suggest that while the Task C model provides reasonable probabilistic estimates for sensitivity-prioritized case finding, it should be interpreted as an initial screening tool rather than a stand-alone diagnostic instrument. The relatively low specificity implies a substantial false-positive burden, so caution is warranted when interpreting the absolute probability values. Further calibration may be necessary before clinical deployment or for use in high-threshold decision-making (Table 4).

**Table 4:** Calibration Assessment of the Evaluated Models in Task C.

Model	Brier Score	Hosmer-Lemeshow Statistic	df	p-value	Number of Groups
Stacking	0.2482	29.5320	8	0.0003	10
SVM	0.2518	22.8154	8	0.0036	10
GBDT	0.2450	23.7266	8	0.0025	10
LightGBM	0.2446	20.5544	8	0.0084	10
XGBoost	0.2455	21.7112	8	0.0055	10
AdaBoost	0.2497	23.6135	8	0.0027	10
LR	0.2487	27.1844	8	0.0007	10

#### Analysis of ROC and PR curves in opportunistic screening

**Fig. 5:**
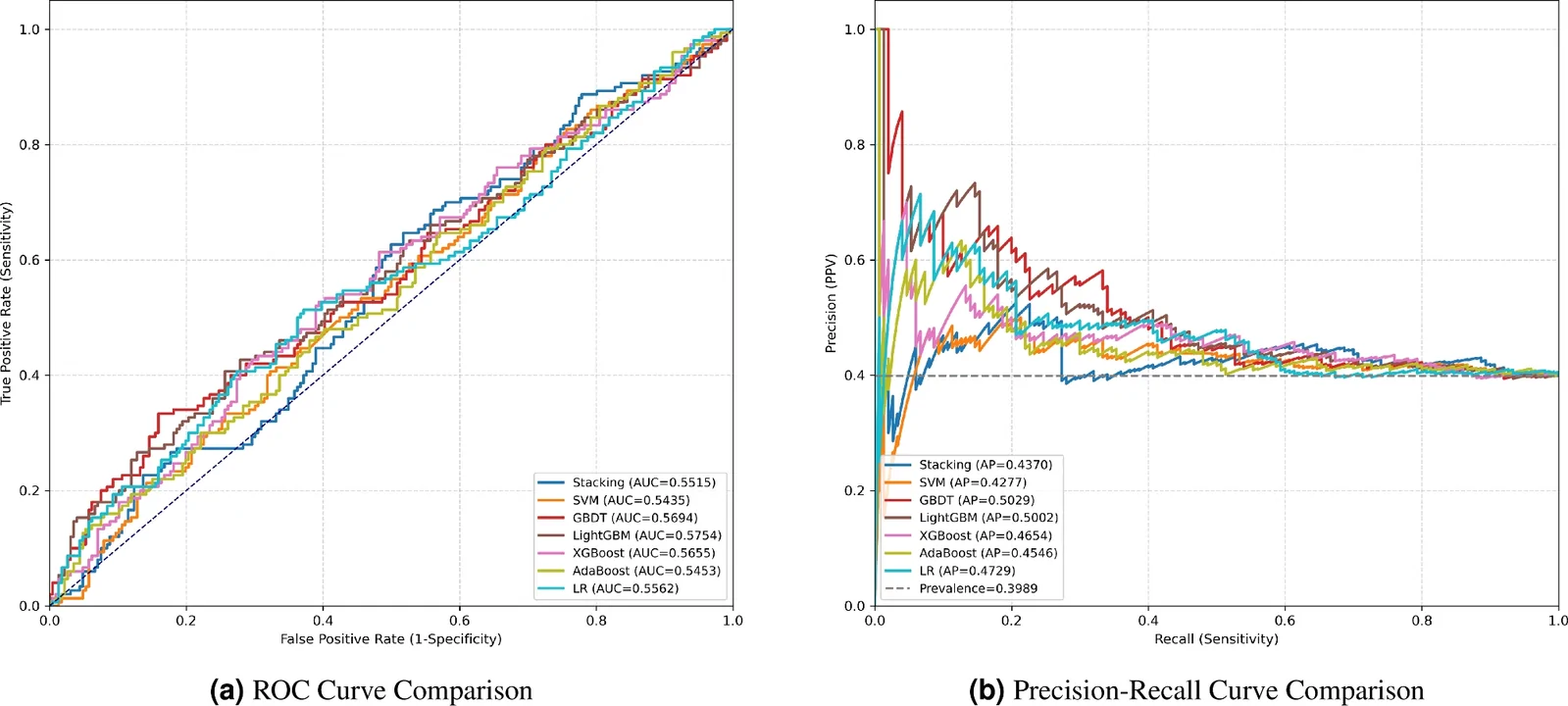
Performance Analysis in the Opportunistic Screening Scenario (Task C). (**a**) Receiver Operating Characteristic (ROC) curves of the evaluated models. In the absence of biochemical markers, all models showed modest discriminative ability; LightGBM achieved the highest AUC (0.5754), whereas the optimized Stacking model achieved an AUC of 0.5515. (**b**) Precision–Recall (PR) curves illustrating the trade-off between precision and recall under the non-invasive screening setting. The prevalence baseline was 0.3989. GBDT achieved the highest Average Precision (AP = 0.5029), followed closely by LightGBM (AP = 0.5002), while the Stacking model achieved an AP of 0.4370.

To visually assess model discrimination and classification trade-offs under the feature-constrained Task C setting, Receiver Operating Characteristic (ROC) and Precision–Recall (PR) curves were generated (Fig. 5).

As shown in Fig. 5a, the ROC curves were close to the diagonal reference line, indicating that the discriminative ability of all models was modest when only non-invasive variables were available. LightGBM achieved the highest AUC (0.5754), followed by GBDT (0.5694) and XGBoost (0.5655), whereas the optimized Stacking model achieved an AUC of 0.5515. These results suggest that non-invasive demographic, lifestyle, symptom-related, and medical-history variables contain limited but non-negligible risk information for diabetes screening.

The PR curves further illustrate the challenge of opportunistic screening in this feature-restricted scenario (Fig. 5b). The prevalence baseline was 0.3989, and most models showed only moderate improvement over this baseline. GBDT achieved the highest Average Precision (AP = 0.5029), followed closely by LightGBM (AP = 0.5002), whereas the Stacking model achieved an AP of 0.4370. Although the Stacking model did not dominate the ROC or PR curves, it maintained high sensitivity at the selected screening threshold, supporting its role as a preliminary screening stratification tool rather than a stand-alone diagnostic model.

Overall, the ROC and PR analyses indicate that Task C is a challenging non-invasive screening scenario. The modest AUC and AP values suggest that non-invasive variables alone are insufficient for definitive diabetes classification. However, they may still be useful for identifying individuals who should be prioritized for subsequent confirmatory biochemical testing.

#### Operating threshold selection and trade-off analysis

To align with the clinical objective of opportunistic screening, the operating thresholds were not fixed at the default value of 0.5. Instead, a sensitivity-prioritized thresholding strategy was adopted. For each model, the decision threshold was selected from training-set out-of-fold predictions by identifying an operating point that satisfied the predefined sensitivity requirement (Recall *≥ 0.90*). The selected threshold was then fixed and applied to the independent held-out test set.

For the optimized Stacking model in Task C, the operating threshold was determined as $p_t=0.4596$. At this threshold, the model achieved a Recall of 0.9267, indicating that a large proportion of labeled diabetic cases were flagged under the non-invasive screening setting. Compared with several single models that required lower thresholds to reach similar sensitivity levels, the Stacking model maintained a relatively higher operating threshold while achieving the highest Precision (0.4088), Specificity (0.1106), and F1-score (0.5673) among the evaluated models in Task C.

This threshold choice inevitably produced a low specificity, reflecting a substantial false-positive burden. However, this trade-off is consistent with the intended role of Task C as an initial opportunistic screening tool rather than a stand-alone diagnostic model. In this setting, false-positive individuals can be referred for subsequent confirmatory biochemical testing, whereas false negatives may result in missed opportunities for early detection. Therefore, the selected threshold prioritizes sensitivity while maintaining the most favorable threshold-dependent balance among the compared models in the non-invasive screening scenario.

### Subgroup analysis: performance consistency across demographics

To evaluate the demographic robustness of the proposed Stacking framework in the Task C non-invasive opportunistic screening scenario, subgroup analyses were conducted across gender and age strata. The objective was to examine whether the model could maintain screening-oriented performance across different demographic groups while identifying potential disparities in threshold-dependent metrics (Table 5).

**Table 5:** Subgroup Analysis of Model Performance by Gender and Age.

Group	Subgroup	Count	AUC	Recall	Precision	F1-Score
Gender	Male	195	0.5852	0.9710	0.3661	0.5317
	Female	181	0.5320	0.8889	0.4586	0.6050
Age	< 50 y/o	160	0.5654	0.9315	0.4755	0.6296
	*≥* 50 y/o	216	0.5526	0.9221	0.3604	0.5182

In the gender-stratified analysis, the male subgroup achieved a higher AUC than the female subgroup (0.5852 vs. 0.5320) and exhibited higher Recall (0.9710 vs. 0.8889), indicating that most male cases were flagged under the sensitivity-prioritized threshold. However, males showed lower Precision (0.3661 vs. 0.4586) and F1-score (0.5317 vs. 0.6050), reflecting a greater false-positive burden in this subgroup.

For age stratification, the model achieved similar AUCs in the younger (*<50* years, *N=160*) and older (*≥ 50* years, *N=216*) groups (0.5654 vs. 0.5526). Recall remained high in both age groups (0.9315 and 0.9221, respectively), consistent with the model’s objective of maximizing case detection in opportunistic screening. Nevertheless, the younger group showed higher Precision (0.4755) and F1-score (0.6296) than the older group (0.3604 and 0.5182), suggesting that the false-positive burden may be greater among older participants.

Overall, the subgroup analysis suggests that the Task C Stacking model generally preserved high sensitivity across demographic strata, although its discriminative ability and positive predictive performance remained modest in the non-invasive, feature-constrained setting. These findings support its use as a preliminary screening stratification tool while highlighting the need for confirmatory biochemical testing after positive screening results.

### Clinical utility assessment

**Fig. 6:**
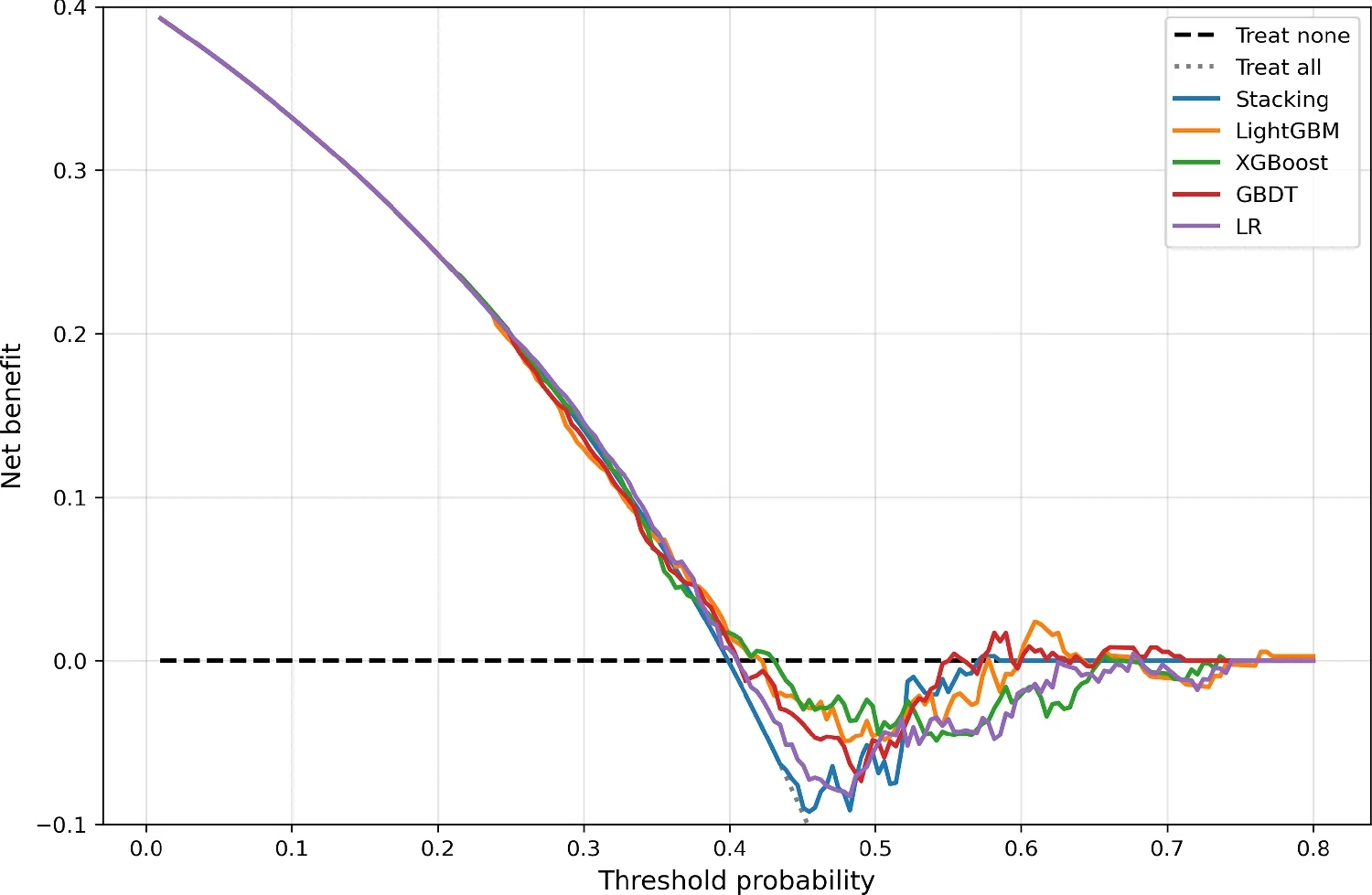
Decision curve analysis (DCA).

To evaluate the clinical utility of the evaluated models under different risk tolerance levels, Decision Curve Analysis (DCA) was performed for the Task C opportunistic screening scenario (Fig. 6). At low threshold probabilities, the Treat-All strategy showed a high net benefit, reflecting the sensitivity-prioritized nature of preliminary screening. As the threshold probability increased, the net benefit of Treat-All declined rapidly and approached zero at approximately $p_t = 0.40$.

Across the model-based curves, the net benefits were generally modest, consistent with the limited discriminative ability observed in the ROC and PR analyses under the non-invasive feature setting. The evaluated models showed broadly similar decision-curve patterns, and the Stacking model did not consistently dominate the single models across the full threshold range. Nevertheless, its curve remained comparable to those of the evaluated single learners, suggesting that the Task C Stacking model may provide practical value mainly within the lower-threshold screening range.

At higher threshold probabilities, particularly beyond approximately $p_t = 0.45$, the net benefit of most models became close to zero or negative. This indicates that the non-invasive Task C model is not suitable for high-threshold decision-making or definitive diagnosis. Overall, the DCA results support the intended role of Task C as a preliminary opportunistic screening tool for identifying individuals who may benefit from confirmatory biochemical testing, rather than as a stand-alone diagnostic model.

## Model explainability and screening-oriented simulation

Although the heterogeneous Stacking architecture improves screening performance, it lacks direct decision-making transparency. To improve interpretability and characterize the associational patterns underlying the model outputs, we applied the SHAP (SHapley Additive exPlanations) framework.

It is important to note that SHAP values quantify associations with the model-estimated screening probability rather than biological causation. Therefore, the subsequent *in silico* simulations should be interpreted as model-based input modification analyses derived from cross-sectional data. These analyses were designed to support opportunistic screening and case-finding by identifying modifiable factors associated with higher screening probabilities.

### SHAP analysis of modifiable factors

**Fig. 7:**
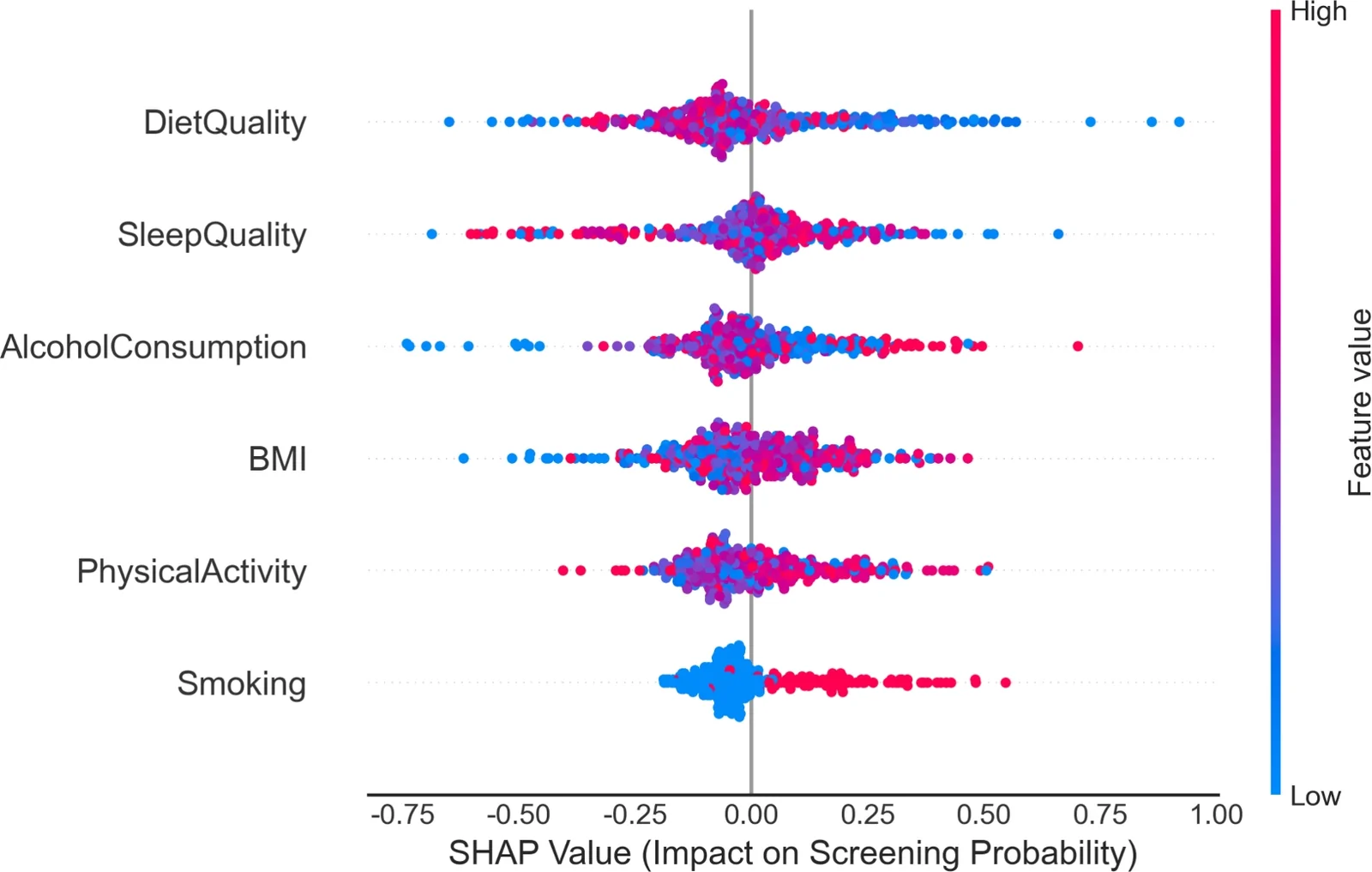
SHAP Summary of Modifiable Features for Task C Opportunistic Screening.

To focus the interpretability analysis on actionable variables, we visualized SHAP values for six predefined modifiable features: *Diet Quality*, *Sleep Quality*, *Alcohol Consumption*, *Physical Activity*, *BMI*, and *Smoking*. As shown in Figure 7, these features are ranked according to their mean absolute SHAP values, reflecting their relative contributions to the model-estimated screening probability within the modifiable feature space.

#### Relative importance of modifiable factors

As illustrated in the SHAP summary plot (Figure 7), *Diet Quality*, *Sleep Quality*, and *Alcohol Consumption* emerged as the top three contributors among the modifiable factors. These self-reported behavioral variables showed greater contribution to the screening probability than *Physical Activity*, *BMI*, and *Smoking*, which ranked lower in the modifiable feature hierarchy.

#### Directionality of model-based associations

The SHAP value distribution further illustrates the direction of each feature’s association with the model-estimated screening probability:

- *Protective lifestyle indicators:* For *Diet Quality*, *Sleep Quality*, and *Physical Activity*, higher feature values were generally associated with negative SHAP values, indicating an inverse association with the model-estimated screening probability.
- *Risk-associated factors:* For *Alcohol Consumption*, *BMI*, and *Smoking*, higher feature values tended to contribute positively to the model output, suggesting an association with higher screening probability.

These directional patterns should be interpreted as model-derived associations within the cross-sectional Task C case-finding setting rather than causal effects.

### *In silico* screening probability simulation of modifiable factors

To examine how hypothetical modifications of modifiable factors influence the model-estimated screening probability, we conducted an *in silico* input modification analysis. Predefined boundary constraints were used to maintain plausibility during these hypothetical modifications:

- *BMI Adjustment:* The simulated reduction was applied exclusively to samples with a baseline BMI *> 24* (overweight/obese). For these individuals, the simulated BMI value was set to 90% of the baseline BMI, approximating a 10% body-weight reduction scenario under unchanged height.
- *Behavioral Variable Modification:* For non-BMI modifiable behavioral variables, the simulated value was set to the empirically observed favorable extreme within the dataset. Specifically, risk-associated behaviors, such as *Smoking* and *Alcohol Consumption*, were set to their minimum observed values, whereas protective behavioral indicators, such as *Diet Quality*, *Sleep Quality*, and *Physical Activity*, were set to their maximum observed values.

#### Implementation of the “Top-3” screening-oriented simulation strategy

**Fig. 8:**
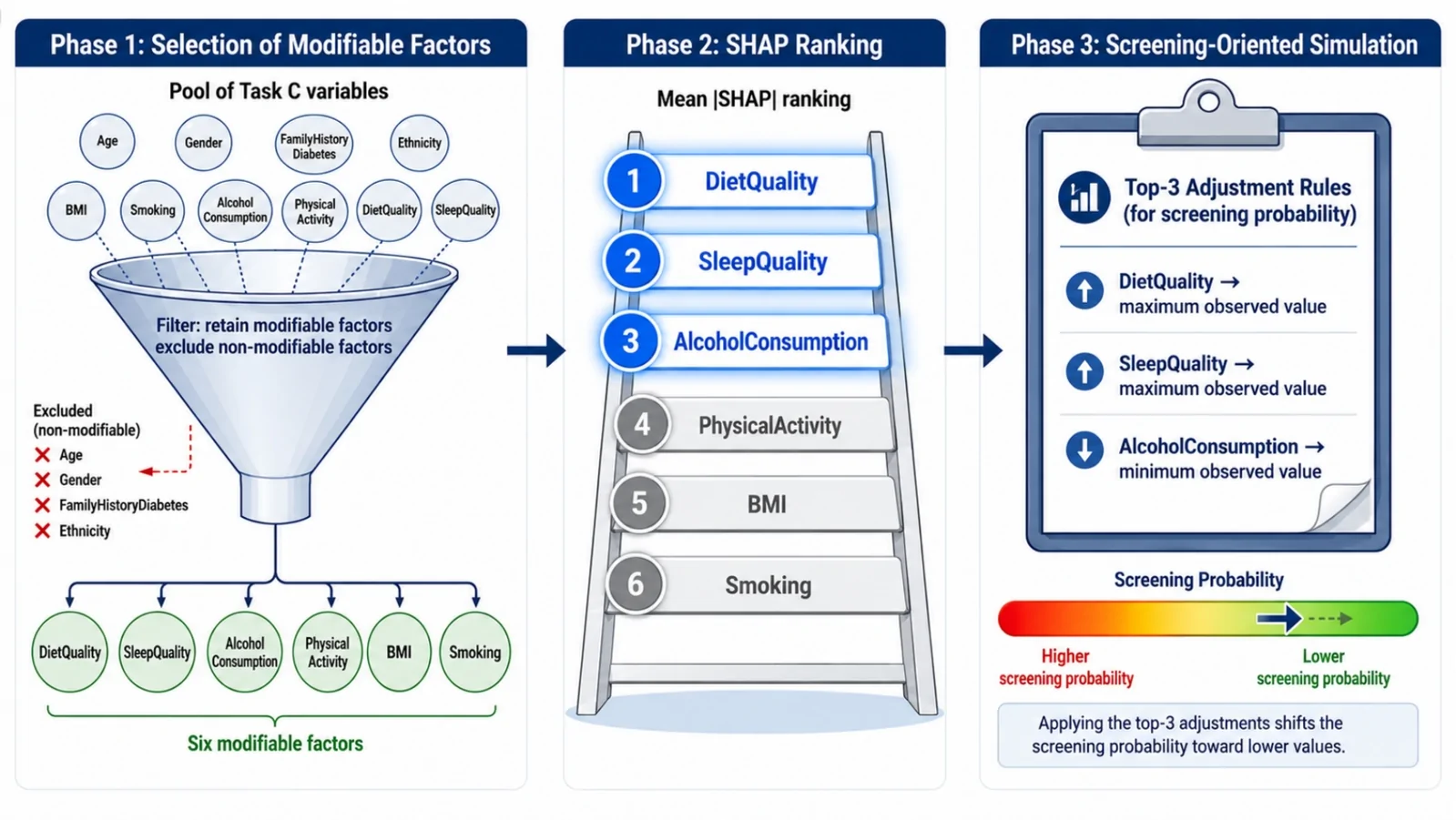
Top-3 Screening-Oriented Simulation Workflow. The workflow consists of three stages: selection of six modifiable factors from the Task C feature space, SHAP-based ranking according to mean absolute SHAP values, and hypothetical input modification of the top three factors. The top-ranked modifiable factors were *DietQuality*, *SleepQuality*, and *AlcoholConsumption*. Protective factors were set to their maximum observed values, whereas risk-associated factors were set to their minimum observed values. The simulated probability shift reflects changes in model-estimated screening probability and does not imply causal intervention effects.

To systematically evaluate the potential impact of targeted hypothetical adjustments, we implemented a “Top-3” feature modification pipeline. The algorithmic execution mechanism, as schematically illustrated in Figure 8, comprises three sequential stages:

- *Static feature filtering:* The system automatically identifies and excludes non-modifiable features (e.g., Age, Gender, and Genetic Background), restricting the simulation targets exclusively to the modifiable behavioral space.
- *SHAP-based contribution ranking:* The remaining modifiable features are ranked in descending order according to mean absolute SHAP values. This step identifies the top three features contributing most strongly to the model-estimated screening probability.
- *Targeted input modification:* Hypothetical modifications are applied only to these three highest-ranked features, while all other variables retain their baseline values. This approach estimates the change in model-estimated screening probability associated with focused input modifications.

**Fig. 9:**
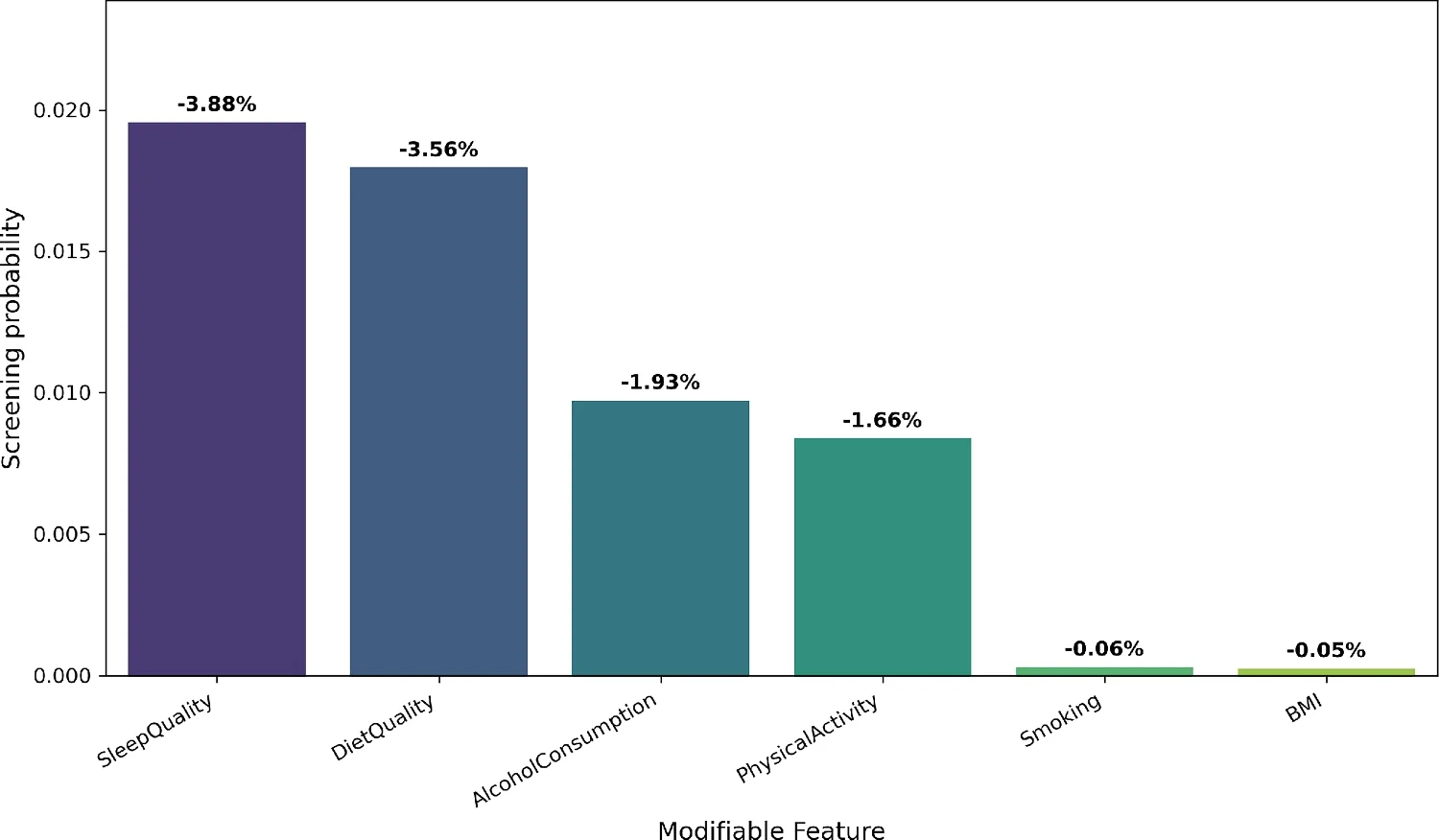
Ranking of Simulated Screening Probability Reductions by Modified Feature. Hypothetical modifications of SleepQuality and DietQuality yielded the largest average reductions in model-estimated screening probability, followed by AlcoholConsumption and PhysicalActivity, whereas BMI and Smoking showed minimal changes.

**Fig. 10:**
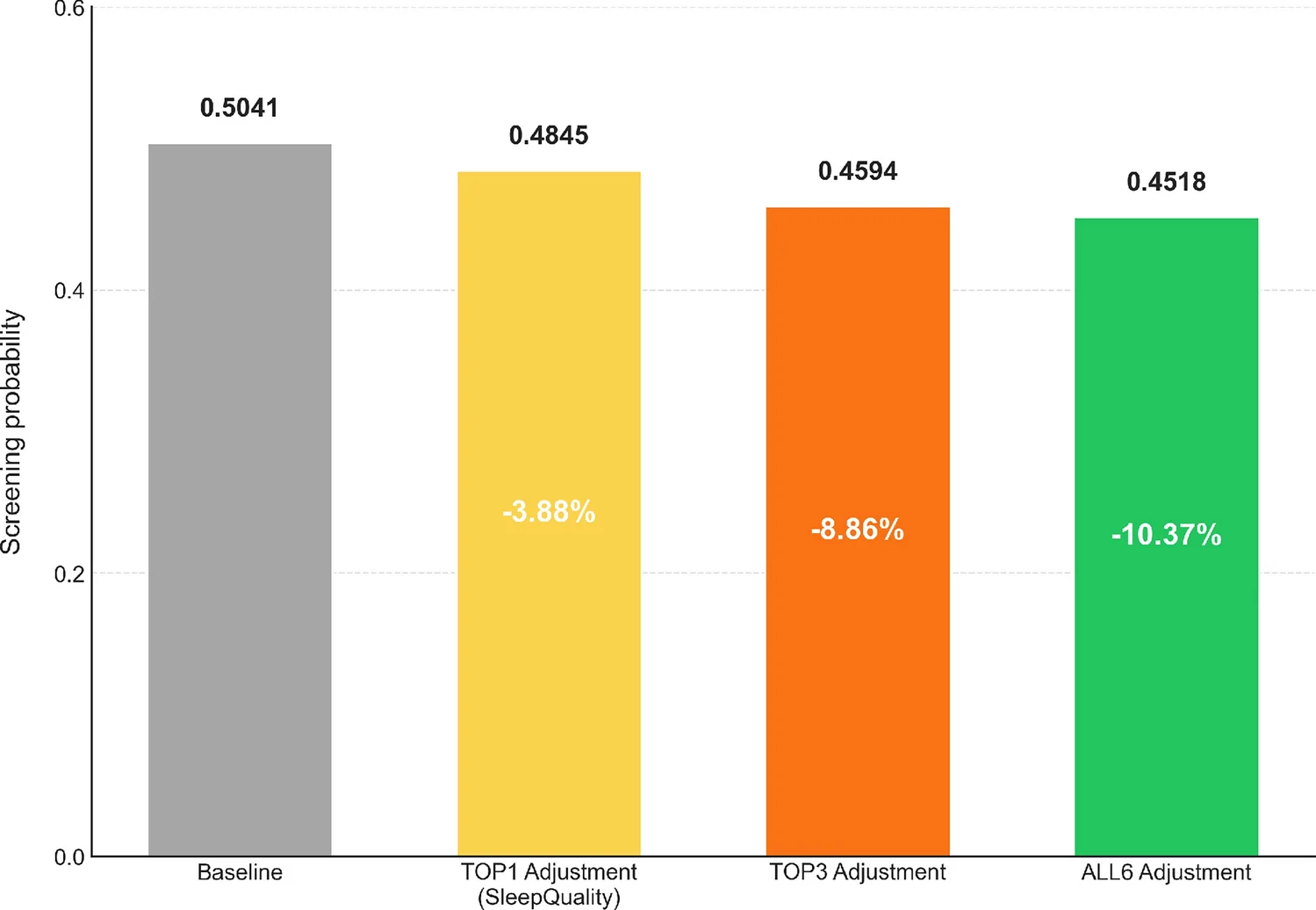
Comparison of Simulated Screening Probability Reductions Under Different Adjustment Strategies. The baseline mean model-estimated screening probability was 0.5041. Single-factor adjustment of SleepQuality reduced the screening probability to 0.4845, while the TOP3 and ALL6 strategies further reduced it to 0.4594 and 0.4518, respectively. Percentage labels indicate relative reductions from baseline.

### Simulated changes in model-estimated screening probability

To explore the potential interpretability of modifiable non-invasive factors, a model-based counterfactual simulation was conducted among individuals classified as high-risk by the Task C Stacking model. High-risk individuals were defined according to the selected operating threshold ($p_t = 0.4596$). For each modifiable factor, a predefined hypothetical adjustment was applied to the feature values, and the adjusted feature vectors were re-evaluated by the trained Stacking model. The resulting change in model-estimated screening probability was quantified as the absolute probability reduction. This analysis should be interpreted as a model-based simulation rather than evidence of causal intervention effects.

#### Comparison of simulated reductions across single factors

As shown in Fig. 9, modifying *SleepQuality* produced the largest average reduction in model-estimated screening probability, with an absolute reduction of approximately 0.0196 and a relative reduction of 3.88%. This was followed by *DietQuality*, which showed an absolute reduction of approximately 0.018 and a relative reduction of 3.56%. *AlcoholConsumption* and *PhysicalActivity* showed smaller reductions, corresponding to relative decreases of 1.93% and 1.66%, respectively. In contrast, modifying *Smoking* and *BMI* produced minimal changes in model-estimated screening probability in this simulation.

These findings suggest that, within the learned Task C Stacking model, sleep quality and diet quality contributed most strongly to the simulated reduction in model-estimated screening probability among high-risk individuals. However, because the analysis modifies model inputs rather than observing real-world behavioral changes, the results should be interpreted as hypothesis-generating and not as causal estimates of lifestyle intervention effects.

#### Comparison of combined adjustment strategies

We further evaluated the simulated impact of combining multiple hypothetical feature adjustments, as illustrated in Fig. 10. The baseline mean model-estimated screening probability among high-risk individuals was 0.5041.

- *Single-factor adjustment (TOP-1):* Modifying the highest-ranked single factor, *SleepQuality*, reduced the mean model-estimated screening probability from 0.5041 to 0.4845, corresponding to a relative reduction of 3.88%.
- *Targeted adjustment (TOP-3):* When the three most influential modifiable factors were adjusted simultaneously, the mean model-estimated screening probability decreased to 0.4594, corresponding to a relative reduction of 8.86%.
- *Comprehensive adjustment (ALL-6):* Simultaneously modifying all six predefined modifiable factors further reduced the mean model-estimated screening probability to 0.4518, corresponding to a relative reduction of 10.37%.
- *Relative efficiency:* The TOP3 adjustment achieved approximately 85.5% of the relative reduction observed under the ALL6 simulation ($8.86\%/10.37\%$), suggesting that a targeted strategy may capture most of the model-estimated probability reduction while requiring fewer behavioral modifications.

These results indicate that the simulated reduction in model-estimated screening probability increased as the number of adjusted modifiable factors expanded. However, the additional gain from ALL6 compared with TOP3 was relatively small, supporting the potential practicality of a focused TOP3 strategy. Because this analysis is based on hypothetical feature modifications, the results should be interpreted as model-based counterfactual simulations rather than causal estimates of real-world intervention effects.

### Statistical evaluation of simulated probability shifts

To assess whether the simulated reductions in model-estimated screening probability under the Top-3 strategy were statistically significant, we analyzed the high-risk cohort (*N=340*) from the Task C test set, defined by baseline model-estimated screening probabilities exceeding the operating threshold ($p_t = 0.4596$). A paired t-test was performed to compare model-estimated screening probabilities before and after the hypothetical feature adjustments.

#### Significance of the algorithmic shift

**Fig. 11:**
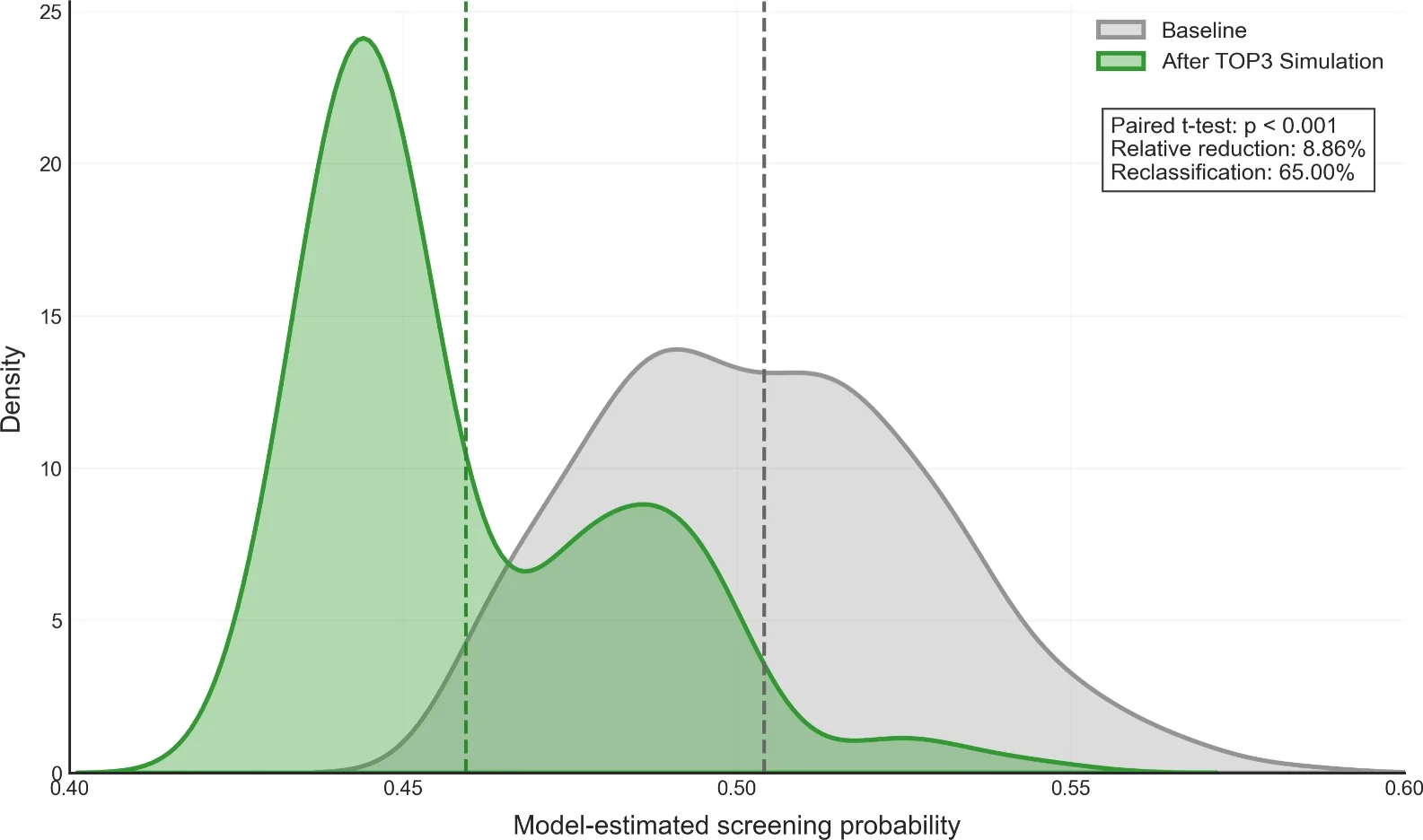
Probability Density Distributions Pre- and Post-Simulation for High-Risk Cohort. The statistically significant downward shift in model-estimated screening probabilities (*p < 0.001*) demonstrates a consistent reduction in the model’s output following hypothetical Top-3 feature modifications.

Figure 11 illustrates the probability density distributions of the high-risk cohort before (gray) and after (green) Top-3 simulation. The mean model-estimated screening probability decreased from 0.5041 to 0.4594 following the adjustments. The paired t-test indicated a statistically significant reduction (*t = 32.73*, *p < 0.001*), indicating a consistent and measurable shift in the model output following the targeted feature modifications.

#### Reclassification rate after top-3 simulation

To quantify the screening-level implication of the algorithmic adjustments, we examined the reclassification rate relative to the screening threshold. Following the Top-3 feature modifications, 65.00% (221/340) of high-risk individuals were reclassified below the operating threshold ($p_t \le 0.4596$). This demonstrates that, under the favorable-extreme simulation setting, hypothetical modification of a limited number of key lifestyle and behavioral features could shift the model-estimated screening classification for a substantial subset of high-risk individuals.

### Case study: individual-level screening probability simulation

To illustrate the application of the simulated adjustment strategy at the individual level, we selected a representative high-risk sample from the held-out test set and evaluated the model-estimated screening probability trajectory under different hypothetical adjustment scopes.

#### Baseline algorithmic assessment

**Fig. 12:**
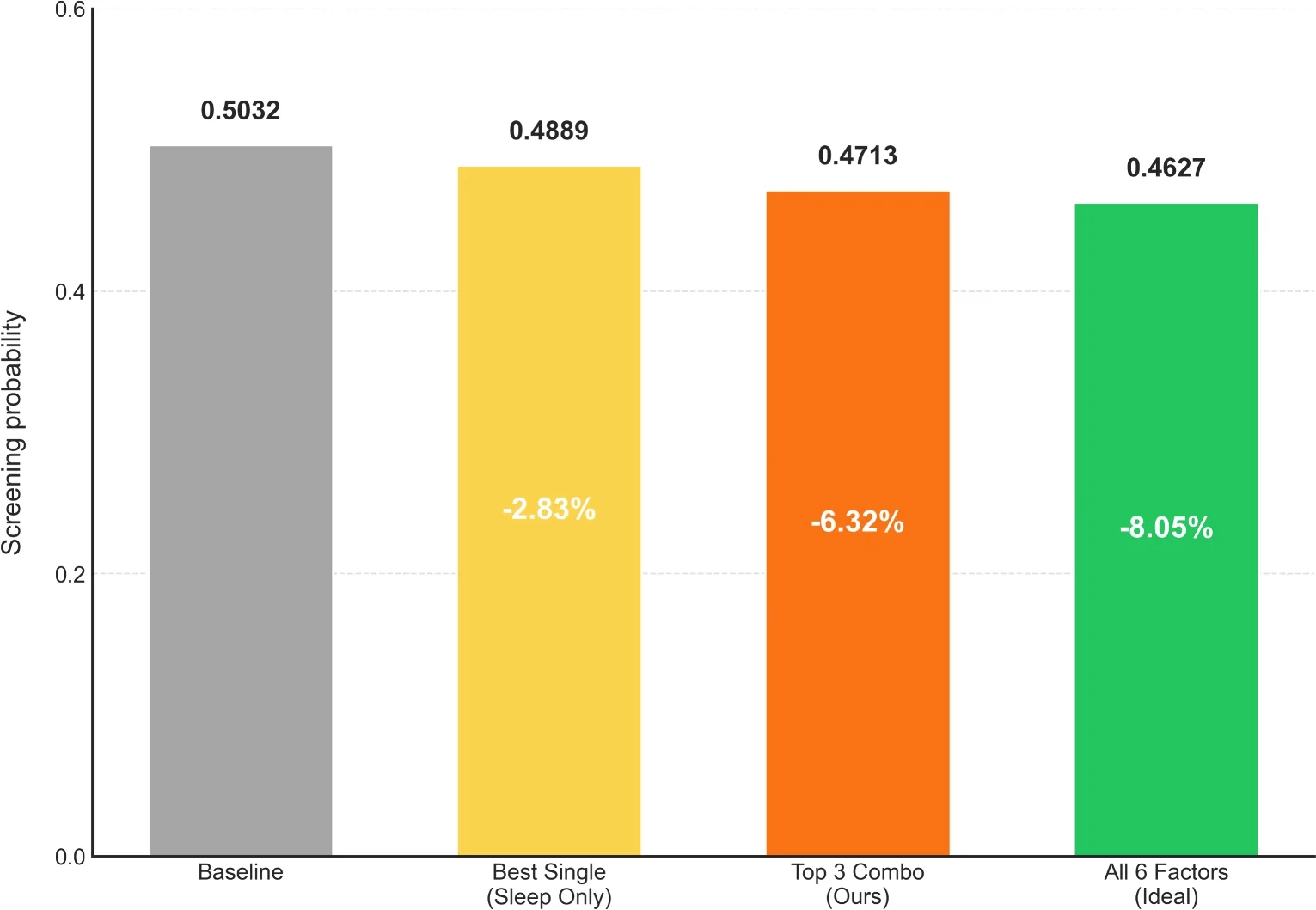
Trajectory of Model-Estimated Screening Probability for a Representative High-Risk Individual under Simulated Modifications. The All-6 strategy produced the lowest model-estimated screening probability, whereas the Top-3 strategy achieved most of the simulated reduction with fewer hypothetical feature modifications.

For the selected individual, the Task C Stacking model estimated a baseline screening probability of 0.5032 (Fig. 12), which was above the operating threshold used for opportunistic screening. This sample was therefore classified as high-risk under the sensitivity-prioritized screening setting.

#### Screening probability trajectory under different adjustment scopes

As shown in Fig. 12, modifying only the highest-ranked factor, *SleepQuality*, reduced the model-estimated screening probability from 0.5032 to 0.4889, corresponding to a relative reduction of 2.83%. However, this single-factor adjustment produced only a modest probability shift.

When the Top-3 strategy was applied, the model-estimated screening probability further decreased to 0.4713, corresponding to a relative reduction of 6.32%. A comprehensive adjustment of all six predefined modifiable factors reduced the model-estimated screening probability to 0.4627, corresponding to a relative reduction of 8.05%.

These results suggest that, for this individual, combining multiple modifiable non-invasive factors produced a larger simulated reduction in model-estimated screening probability than modifying a single factor alone. The additional improvement from the All-6 strategy over the Top-3 strategy was relatively small, indicating that a targeted Top-3 approach may capture most of the model-estimated probability reduction while requiring fewer hypothetical adjustments. As with the cohort-level simulation, this case analysis should be interpreted as a model-based counterfactual illustration rather than evidence of causal intervention effects.

## Discussion

This study investigated a cost-effective approach for opportunistic diabetes screening using exclusively non-invasive features. In the Task C scenario, the optimized Stacking model achieved a high Recall of 0.9267 while excluding biochemical markers, demonstrating its ability to prioritize case detection under a sensitivity-driven screening objective. However, its discriminative performance remained modest, with an AUC of 0.5515 and a Brier Score of 0.2482. The relatively low specificity of 0.1106 further indicates a substantial false-positive burden. These findings suggest that non-invasive variables alone provide limited but potentially useful risk information for preliminary screening.

The findings suggest that the heterogeneous Stacking ensemble can integrate demographic, lifestyle, medical-history, and symptom-related predictors to support preliminary screening stratification. Importantly, the model should not be interpreted as a stand-alone diagnostic tool. Rather, its value lies in identifying individuals who may benefit from subsequent confirmatory biochemical testing. In addition, the simulated adjustment analyses showed that targeted hypothetical modifications of key modifiable factors were associated with reductions in model-estimated screening probability among high-risk individuals, providing a hypothesis-generating basis for individualized health management guidance.

### Strategic optimization and public health implications

Two practical implications emerge from the model evaluation and simulation analyses:

- *Two-stage sequential screening:* Given the low specificity of Task C, a hierarchical screening strategy is more appropriate than direct diagnosis. The non-invasive Stacking model can be used as a broad initial triage tool to prioritize sensitivity and reduce the likelihood of missed labeled cases. Individuals flagged as high-risk can then undergo confirmatory biochemical testing. This two-stage structure makes the false-positive burden more manageable while preserving the public health value of early case detection.
- *Targeted behavioral adjustment:* The model-based simulation suggested that reductions in model-estimated screening probability were not evenly distributed across modifiable factors. Sleep quality and diet quality produced the largest single-factor probability reductions, while combining the top three modifiable factors achieved a relative reduction of 8.86%. Compared with the comprehensive six-factor adjustment, which achieved a relative reduction of 10.37%, the Top-3 strategy captured approximately 85.5% of the simulated benefit. This suggests that a focused strategy targeting a small number of key behavioral factors may offer a pragmatic balance between feasibility and model-estimated screening probability reduction. Nevertheless, these findings should be interpreted as model-based counterfactual simulations rather than causal intervention effects.

### Integration with medical knowledge-augmented modeling

While our data-driven SHAP framework provides transparency for ensemble predictions, recent advancements highlight the necessity of integrating clinical domain knowledge to further constrain algorithmic explanations. As emphasized in recent literature, purely data-driven hybrid models can suffer from the curse of dimensionality, which can be mitigated by incorporating physiological constraints^7^. Furthermore, augmenting knowledge with SHAP-based analysis^8^ or Large Language Models (LLMs)^19^ has proven effective in ensuring that algorithmic attributions remain biologically plausible. Future iterations of our framework will explore combining SHAP-derived rankings with expert-defined knowledge graphs to prevent the prioritization of spurious correlations, thereby enhancing the translational robustness of the targeted Top-3 strategy.

### Limitations and future prospects

While this study provides preliminary evidence for the algorithmic feasibility of non-invasive screening, certain limitations warrant attention. First, given the cross-sectional nature of the training data, this framework is rigorously defined as an instrument for opportunistic screening rather than longitudinal risk prediction. Consequently, the simulated probability shifts are based on hypothetical input modifications derived from observational correlations. These associational findings do not guarantee actual biological causation or clinical efficacy. Future work intends to conduct prospective cohort studies to empirically evaluate true longitudinal outcomes in real-world settings.

Second, the non-invasive Task C prioritizes sensitivity at the expense of specificity, explicitly mandating a secondary biochemical confirmation step to manage false positives. Third, the model was trained and evaluated on a single cohort dataset (*N = 1879*). Consequently, the generalizability of the findings to broader populations remains unverified. Subsequent research should prioritize external validation across independent, multi-center cohorts to assess the cross-population robustness of the opportunistic screening thresholds. Finally, incorporating longitudinal multi-modal data will be essential to further capture the dynamic evolution of metabolic profiles over time.

## Conclusion

In conclusion, this study developed a heterogeneous Stacking ensemble framework for the non-invasive opportunistic screening of diabetes. In the Task C scenario, the proposed Stacking model achieved a high Recall of 0.9267 using exclusively non-invasive demographic, lifestyle, medical-history, and symptom-related features. Although its discriminative ability remained modest (AUC = 0.5515), the model provided a sensitivity-oriented preliminary screening tool for identifying individuals who may benefit from confirmatory biochemical testing.

Furthermore, the model-based screening simulations indicated that hypothetical modification of a prioritized subset of modifiable features was associated with reductions in model-estimated screening probability among individuals classified as high-risk. The Top-3 strategy achieved a relative reduction of 8.86% in model-estimated screening probability, capturing approximately 85.5% of the reduction observed under the comprehensive six-feature adjustment. While these simulated findings do not establish clinical causality, they provide a hypothesis-generating framework for identifying potentially relevant behavioral factors in opportunistic screening.

Future research should focus on external validation in independent and multi-center cohorts, as well as prospective longitudinal studies to assess whether the identified model-derived patterns can inform clinically meaningful screening and prevention strategies.

## Data Availability

The datasets generated and/or analysed during the current study are available in the following repository.[https://www.kaggle.com/datasets/rabieelkharoua/diabetes-health-dataset-analysis].
